# Collaborative Care Model for Patients With Opioid Use Disorder and Mental Illness

**DOI:** 10.1001/jamanetworkopen.2024.49012

**Published:** 2024-11-26

**Authors:** Katherine E. Watkins, Rebecca Weir, Lia Pak, Beth Ann Griffin, Amber Griffo, Allison Trosclair Sutherland, Colleen M. McCullough, Lisa S. Meredith, Michael Schoenbaum, Miriam Komaromy, Valerie Carrejo, Karen Chan Osilla

**Affiliations:** 1 RAND Corporation, Santa Monica, California; 2 RAND Corporation, Arlington, Virginia; 3 RAND Corporation, Boston, Massachusetts; 4 Department of Family and Community Medicine, University of New Mexico, Albuquerque; 5 First Choice Community Healthcare, Albuquerque, New Mexico; 6 Division of Services and Intervention Research, National Institute of Mental Health, Bethesda, Maryland; 7 Division of General Internal Medicine, Department of Medicine, Chobanian and Avedisian School of Medicine, Boston University, Boston, Massachusetts; 8 Department of Psychiatry and Behavioral Sciences, Stanford University School of Medicine, Palo Alto, California

## Abstract

This cohort study uses data from a randomized clinical trial for opioid use disorder (OUD) co-occurring with mental illness to examine patient characteristics associated with collaborative care model engagement and fidelity.

## Introduction

Opioid use disorders (OUDs) remain undertreated, particularly when co-occurring with mental illness.^1,2^ The collaborative care model (CoCM), an evidence-based approach for integrating behavioral health treatment in primary care, offers a potential solution, but the extent to which the CoCM can engage high-risk populations with fidelity in community settings is poorly understood. The present study used data from a CoCM trial for OUD co-occurring with mental illness to examine patient characteristics associated with CoCM engagement and fidelity.

## Methods

This cohort study used data from a randomized clinical trial of the CoCM in 14 low-resourced, primary care clinics in New Mexico.^3^ The study was approved by RAND’s institutional review board and followed the STROBE reporting guideline for cohort studies; all participants provided written informed consent. Participants were adults with probable OUD and co-occurring mental illness assigned to the CoCM between January 1, 2021, and December 31, 2023. The CoCM included community health workers as care managers, addiction-certified psychiatric consultants, and primary care clinicians, supported by a caseload tracking tool.^4^

Engagement and fidelity data came from the caseload tracking tool; demographic and clinical characteristics and social determinants associated with illness severity and access to care came from the participant baseline interview. Engagement was defined as having an intake encounter with the care manager. Fidelity was defined as having at least 2 care manager encounters, at least 2 assessments of OUD and mental health symptom severity, and the treatment plan reviewed by the psychiatric consultant within the 6-month intervention period.

We report on the association of patient demographics, mental health, substance use, and social determinants with CoCM engagement and fidelity. We used SAS, version 9.4 for all data management tasks and Stata, version 17 to run statistical tests and generate tables (eMethods in Supplement 1).

## Results

Of 369 patients aged 18 years or older assigned to the CoCM (mean [SD] age, 39.9 [12.1] years; 211 [57.2%] women and 158 men [42.8%]), 297 (80.5%) engaged with the CoCM, with a mean (SD) intake encounter duration of 31 (14) minutes (Table). The median number of encounters was 5 (IQR, 1-10). Engagement rates exceeded two-thirds for all subgroups.

**Table.** Engagement and Fidelity to the Collaborative Care Model by Patient Characteristics^a^

Characteristic	Full sample, No. (%) (N = 369)	% (No./total No.) Engaged (n = 369)	Engaged, No. (%) (n = 297)	% (No./total No.) Engaged with fidelity (n = 297)
Age, mean (SD), y	39.9 (12.1)	NA	40.5 (12.3)	NA
Sex				
Female	211 (57.2)	80.6 (170/211)	170 (57.2)	67.1 (114/170)
Male	158 (42.8)	80.4 (127/158)	127 (42.8)	72.4 (92/127)
Ethnicity				
Hispanic	262 (71.0)	79.8 (209/262)	209 (70.4)	67.5 (141/209)
Non-Hispanic	107 (29.0)	82.2 (88/107)	88 (29.6)	73.9 (65/88)
**Mental health factors**				
Suicidal ideation^b^				
No	264 (71.5)	80.3 (212/264)	212 (71.4)	68.9 (146/212)
Yes	105 (28.5)	81.0 (85/105)	85 (28.6)	70.6 (60/85)
Exposure to trauma				
No reported trauma	34 (9.2)	82.4 (28/34)	28 (9.4)	78.6 (22/28)
Trauma, no exposure to interpersonal violence	199 (53.9)	80.9 (161/199)	161 (54.2)	71.4 (115/161)
Trauma with exposure to interpersonal violence	136 (36.9)	79.4 (108/136)	108 (36.4)	63.9 (69/108)
**Substance use factors**				
Type of opioid misuse^b^				
No opioid misuse	227 (61.5)	85.5 (194/227)	194 (65.3)	70.1 (136/194)
Only prescription pain pill misuse	48 (13.0)	77.1 (37/48)	37 (12.5)	81.1 (30/37)
Any heroin or fentanyl use	94 (25.5)	70.2 (66/94)	66 (22.2)	60.6 (40/66)
Stimulant co-use^b^				
No	247 (66.9)	86.2 (213/247)	213 (71.7)	75.6 (161/213)
Yes	122 (33.1)	68.9 (84/122)	84 (28.3)	53.6 (45/84)
Receiving prescribed MOUD^b^				
No	95 (25.7)	75.8 (72/95)	72 (24.2)	72.2 (52/72)
Yes	274 (74.3)	82.1 (225/274)	225 (75.8)	68.4 (154/225)
**Social determinant factors**				
Housing^c^				
Stable	321 (87.0)	81.0 (260/321)	260 (87.5)	71.5 (186/260)
Unstable	48 (13.0)	77.1 (37/48)	37 (12.5)	54.1 (20/37)
Legal trouble^d^				
No	291 (78.9)	81.8 (238/291)	238 (80.1)	70.6 (168/238)
Yes	78 (21.1)	75.6 (59/78)	59 (19.9)	64.4 (38/59)

Abbreviations: MOUD, medication for opioid use disorder; NA, not applicable.

^a^ All percentages listed in parentheses are column percentages. The “% Engaged” and “% Engaged with fidelity” columns display row percentages.

^b^ Past 30 days.

^c^ Past 3 months.

^d^ Past 12 months.

Of the 297 who engaged, 206 (69.4%) received the CoCM with fidelity, with a median of 9 encounters (IQR, 7-12 encounters). Fidelity rates ranged from 53.6% (45 of 84) for those with stimulant co-use to 81.1% (30 of 37) for those who misused prescription pain medication (Table). Effect size differences were less than 0.2 for individuals with suicidal ideation (70.6% [60 of 85] engaged with fidelity), trauma with exposure to interpersonal violence (63.9% [69 of 108]), or legal trouble (64.4% [38 of 59]).

## Discussion

We found that patients with OUD at high risk will engage with care managers and receive the CoCM with fidelity. This finding is significant because individuals with OUD are difficult to engage and retain in treatment, and those with co-occurring disorders often face heightened challenges, resulting in lower access and higher rates of mortality.^2,5^ Our results indicate that the CoCM may offer a solution to the undertreatment of OUD for patients with complex conditions. When community health workers are used, the CoCM may be an efficient approach to address behavioral health professional shortages.

To our knowledge, this study is the first to examine how clinical and social characteristics associated with access to care and illness severity are associated with CoCM fidelity. Fidelity was high even among those with trauma with exposure to interpersonal violence, unstable housing, or legal involvement and among those using heroin or fentanyl or stimulants. This finding is important because the care manager’s case management activities are thought to be critical for positive outcomes.^6^ Patient uptake is an essential component of impact and can illustrate whether the intervention is reaching patients equitably.

Limitations include use of observational data from 1 arm of a randomized clinical trial, which took place in a single state with high OUD rates. Further work is needed to determine whether fidelity to the CoCM is associated with patient outcomes.

## Notes

Supplement 1. **eMethods.** Expanded Description of Inclusion Criteria, Measures and Analytic Approach
eReferences.

Supplement 2.
Data Sharing Statement
