# Supplementary material for: Collaborative Care Model for Patients With Opioid Use Disorder and Mental Illness
Source: JAMA Netw Open. 2024 Nov 26;7(11):e2449012. doi: 10.1001/jamanetworkopen.2024.49012 (PMC11600228; doi:10.1001/jamanetworkopen.2024.49012)
Supplement: Supplement 1. — eMethods. Expanded Description of Inclusion Criteria, Measures and Analytic Approach eReferences. [file jamanetwopen-e2449012-s001.pdf]

Supplementary Online Content

Watkins KE, Weir R, Pak L, et al. Collaborative care model for patients with opioid use disorder and mental illness. *JAMA Netw Open.* 2024;7(11):e2449012. doi:10.1001/jamanetworkopen.2024.49012

eMethods. Expanded Description of Inclusion Criteria, Measures and Analytic Approach
eReferences.

This supplementary material has been provided by the authors to give readers additional information about their work.

© 2024 Watkins KE et al. *JAMA Network Open.*

eMethods. Expanded Description of Inclusion Criteria, Measures and Analytic Approach

Inclusion Criteria. Eligible participants for the Collaboration Leading to Addiction Treatment and Recovery from Other Stresses (CLARO) trial were 18 years or older receiving primary care at a participating study site, had screened positive for OUD with co-occurring PTSD and/or depression; were not already getting medication for opioid use disorder and mental health elsewhere; could speak and understand English or Spanish; and provided informed consent. Patients were excluded if they required immediate medical or psychiatric intervention. The current analysis is limited to participants randomized to the intervention arm from New Mexico.

Data Sources and Variables. This study drew from two data sources. The dependent variables were drawn from a caseload tracking tool used by the CLARO care managers to record patient encounters and assessments. The independent variables were drawn from a baseline assessment that included participant demographics, mental health and substance use clinical status, and social determinants of health. We operationalized engagement as the patient having an initial encounter for an evaluation by the care manager. We operationalized fidelity to the Collaborative Care Model (CoCM) as patients having (1) two or more encounters with the care manager, (2) at least two assessments of OUD and mental health symptom severity, and (3) a review of the patient's treatment plan in a team meeting with the psychiatric consultant within the six-month intervention period.

We report independent variables in four categories, most of which are associated with decreased access to care and changes in morbidity and mortality: demographics, mental health factors, substance use factors, and social determinants of health.[1-6] *Demographic characteristics* include age, sex, ethnicity. *Mental health factors* include an indicator for suicidal ideation in the past 30 days using the Columbia Suicide Severity Rating Scale;[7] trauma with exposure to interpersonal violence indicates harm being intentionally inflicted on one person by another; trauma without exposure to interpersonal violence indicates trauma without intentional harm. *Substance use factors* include type of opioid misuse in the past 30 days, characterized as none, only prescription pain pill misuse, or any heroin/fentanyl use (with or without prescription pain pill misuse); self-reported methamphetamine or other stimulant co-use in the past 30 days; and taking MOUD as prescribed in the past 30 days. S*ocial determinants of health* include experiencing housing instability and legal trouble in the past year (includes awaiting charges, trial, or sentence, or being on probation or parole).

Statistical analyses. We used SAS, version 9.4 for all data management tasks and Stata, version 17 to run statistical tests and generate tables. We counted the number of recorded care manager encounters per patient and calculated the mean duration in minutes per intake encounter. We then examined associations of demographics, mental health, substance use, and social determinants of health with our dependent variables. To do so, we compared those who engaged with those who did not. Among those who engaged, we compared those who met fidelity criteria with those who did not. For each characteristic, we calculated an effect size (ES) difference between the two groups of interest by taking the mean difference between the groups and then dividing by the pooled standard deviation. We used ES statistics to determine whether the sizes of differences were small (i.e., <0.2), medium (i.e., 0.2 to 0.4) or large (i.e., ≥ 0.4) and to avoid performing excessive hypothesis testing.[12] To aid in interpretability, we also report rates of engagement and fidelity by risk factor.

Effect size for fidelity was >0.4 for co-use of stimulants, with those co-using stimulants less likely to receive the intervention with fidelity (53.6% vs. 75.6%). Effect sizes for fidelity were between 0.2-0.4 for type of opioid misuse, housing instability, and age.

© 2024 Watkins KE et al. *JAMA Network Open.*

eReferences.

1. Bohnert ASB, Ilgen MA. Understanding links among opioid use, overdose, and suicide. *N Engl J Med.* 2019;380(1):71-79.
2. Darke S, Williamson A, Ross J, Teesson M. Attempted suicide among heroin users: 12-month outcomes from the Australian Treatment Outcome Study (ATOS). *Drug Alcohol Depend.* 2005;78(2):177-186.
3. Turner BJ, Liang Y. Drug overdose in a retrospective cohort with non-cancer pain treated with opioids, antidepressants, and/or sedative-hypnotics: Interactions with mental health disorders. *J Gen Intern Med.* 2015;30(8):1081-1096.
4. Litz M, Leslie D. The impact of mental health comorbidities on adherence to buprenorphine: A claims based analysis. *Am J Addict.* 2017;26(8):859-863.
5. Clark RE, Baxter JD, Aweh G, et al. Risk factors for relapse and higher costs among Medicaid members with opioid dependence or abuse: Opioid agonists, comorbidities, and treatment history. *J Subst Abuse Treat.* 2015;57:75-80.
6. Zhu Y, Mooney LJ, Yoo C, et al. Psychiatric comorbidity and treatment outcomes in patients with opioid use disorder: Results from a multisite trial of buprenorphine-naloxone and methadone. *Drug Alcohol Depend.* 2021;228:108996.
7. Posner K, Brown GK, Stanley B, et al. The Columbia-Suicide Severity Rating Scale: Initial validity and internal consistency findings from three multisite studies with adolescents and adults. *Am J Psychiatry.* 2011;168(12):1266-1277.
8. Kroenke K, Spitzer RL, Williams JB. The PHQ-9: Validity of a brief depression severity measure. *J Gen Intern Med.* 2001;16(9):606-613.
9. Blevins CA, Weathers FW, Davis MT, Witte TK, Domino JL. The posttraumatic stress disorder checklist for DSM-5 (PCL-5): Development and initial psychometric evaluation. *J Trauma Stress.* 2015;28(6):489-498.
10. Bush K, Kivlahan DR, McDonell MB, Fihn SD, Bradley KA. The AUDIT alcohol consumption questions (AUDIT-C): An effective brief screening test for problem drinking. Ambulatory Care Quality Improvement Project (ACQUIP). Alcohol Use Disorders Identification Test. *Arch Intern Med.* 1998;158(16):1789-1795.
11. Pilkonis PA, Yu L, Dodds NE, et al. Item banks for substance use from the Patient-Reported Outcomes Measurement Information System (PROMIS(®)): Severity of use and positive appeal of use. *Drug Alcohol Depend.* 2015;156:184-192.
12. Cohen J. *Statistical power analysis for the behavioral sciences.* New York, NY: Routledge Academic; 1988.

© 2024 Watkins KE et al. *JAMA Network Open.*
