# Supplementary material for: Collaborative Care Model for Patients With Opioid Use Disorder and Mental Illness
Source: JAMA Netw Open. 2024 Nov 26;7(11):e2449012. doi: 10.1001/jamanetworkopen.2024.49012 (PMC11600228; doi:10.1001/jamanetworkopen.2024.49012)
Supplement: Supplement 2. — Data Sharing Statement [file jamanetwopen-e2449012-s002.pdf]

Data Sharing Statement

Watkins. Collaborative Care Model for Patients With Opioid Use Disorder and Mental Illness. *JAMA Netw Open*. Published November 26, 2024. doi:10.1001/jamanetworkopen.2024.49012

Data
Data available: Yes
Data types: Deidentified participant data, Data dictionary
How to access data: kwatkins@rand.org
When available: beginning date: 03-01-2026

Supporting Documents
Document types: None

Additional Information
Who can access the data: Researchers with approved IRB permissions
Types of analyses: for any purpose
Mechanisms of data availability: with investigator support and after approval of a proposal.
Any additional restrictions: Deidentified survey data will be published to the NIMH Data Archive under Project Number UF1MH121954-01, pending NIH approval of data structures. De-identified data from the caseload tracking tool will be made available on reasonable request to the corresponding author (kwatkins@rand.org). Data aggregation of patients or providers may be used to prevent identifiability by inference.
